# Zearalenone, an Estrogenic Mycotoxin, Is an Immunotoxic Compound 

**DOI:** 10.3390/toxins6031080

**Published:** 2014-03-13

**Authors:** Isis M. Hueza, Paulo Cesar F. Raspantini, Leonila Ester R. Raspantini, Andreia O. Latorre, Silvana L. Górniak

**Affiliations:** 1 Division of Pharmaceutical Sciences, Federal University of São Paulo (UNIFESP–Diadema), Diadema 09913-030, S.P., Brazil; 2 Department of Pathology, School of Veterinary Medicine and Animal Science, University of São Paulo, Pirassununga 05508-270, S.P., Brazil; E-Mails: pcfr@usp.br (P.C.F.R.); esterraspantini@usp.br (L.E.R.R.); andreia.latorre@gmail.com (A.O.L.); gorniak@usp.br (S.L.G.)

**Keywords:** zearalenone, immunotoxicology, endocrine disruptor, estrogen receptor, rats

## Abstract

The aim of this study was to assess the toxic effects of zearalenone (ZEA) on the immune function. Ovariectomised rats were treated daily by gavage with 3.0 mg/kg of ZEA for 28 days. Body weight gain, food consumption, haemotological parameters, lymphoid organs, and their cellularities were evaluated. Moreover, acquired immune responses and macrophage activity were also assessed. ZEA promoted reduction in body weight gain, which is not fully explained by diminished food consumption. Despite no effect on haematological parameters, ZEA caused thymic atrophy with histological and thymocyte phenotype changes and decrease in the B cell percentage in the spleen. With respect to acquired and innate immune responses, no statistically significant differences in delayed-type hypersensitivity were noticed; however, in the ZEA-treated rats, antibody production and peroxide release by macrophages were impaired. The observed results could be related to ZEA activity on ERs; thus, ZEA is an immunotoxic compound similar to estrogen and some endocrine disruptors.

## 1. Introduction

Zearalenone (ZEA), a macrocyclic β-resorcyclic acid lactone, is a non-esteroidal estrogenic mycotoxin produced as a secondary metabolite by numerous species of *Fusarium*, including *F. culmorum*, *F. roseum*, *F.graminearum*, and others [1,2,3]. These fungi are present on almost all continents, and they are known to infest both pre- and post-harvested wheat, barley, rice, maize, and other crops, resulting in the contamination of human foods and animal feed worldwide [4]. In fact, in a study in Europe (EU), where ZEA is prevalent, this mycotoxin was reported to be present in 32% of 5010 samples of mixed cereals [5]. Many reports have revealed the prevalence of high levels of ZEA contamination in cereal crops and other foods worldwide [6,7,8,9]. Moreover, ZEA is a stable compound during both storage/milling and the processing/cooking of food, as indicated by its presence in some grain products, such as bread, beers, and processed feeds [10,11,12].

Natural exposure to ZEA in contaminated food has been implicated as a cause of female reproductive changes as a result of its powerful estrogenic activity: its hormonal action exceeds that of most other naturally occurring non-steroidal estrogens, including soy and clover isoflavones [13]. The hyperestrogenic symptoms of ZEA have been reported in laboratory animals (mice, rats, guinea pigs, hamsters, and rabbits), and domestic species, and prepubertal pigs are the most sensitive species to ZEA toxicosis [2].

The maximum tolerated levels of ZEA for human consumption have been established as 20 μg/kg in food intended for babies and infants, 50 μg/kg in maize-based snacks and breakfast cereals, and 200 μg/kg in unprocessed maize and certain maize products [14]. However, there are reliable case reports of early puberty in girls chronically exposed to ZEA in various regions of the world [15,16,17,18]. 

After oral exposure, ZEA is rapidly absorbed and initially metabolized by the intestinal tissue and hepatocytes; this initiates the biotransformation of the compound into its major biologically active reductive metabolites, α- and β-zearalenol (α- and β-ZOL). The estrogenic activity of ZEA and its metabolites is mediated by their binding affinity to estrogen receptors (ER), and they are as potent as coumestrol and genistein, two endocrine-disrupting phytestrogens. However, unlike the phytestrogens, which bind preferentially to ERβ [19], the affinity of ZEA and its reductive metabolites for ERβ is approximately equal to their affinity for ERα receptors [2]. 

As expected, estrogen-responsive tissues, such as the uterus, mammary gland, bone, brain, and other organs, contain both types ERs, the expression of which is tissue-specific. However, in addition to these tissues, cells of the immune system also have ERs; for instance, Erα is expressed in T cells, NK cells, and macrophages, whereas ERβ is more prominently expressed in B cells and monocytes [20]. Indeed, the immunomodulatory effects of estrogen on cell-mediated responses and antibody production had called much attention due to its role in autoimmune diseases [21,22,23]. Moreover, despite some researches addressed ZEA effect on the immune system of different animal species [24,25,26], few of them employ protocols suggested by regulatory agencies, such as the FDA (Food and Drug Administration), the OECD (Organization for Economic Co-operation and Development), and the NIH (National Institutes of Health), to evaluate the effects of xenobiotics on the innate and acquired immune responses. Thus, the aim of the present study was to evaluate if ZEA, similar to estrogen, interferes on different branches of the immune system in ovariectomized rats. 

## 2. Results

### 2.1. The Dose-Range Study

ZEA causes numerous toxic effects in both domestic and laboratory animals, especially related to the reproductive system. In particular, the strong uterotrophic activity of ZEA is a relevant effect in most mammalian species [27,28,29]. Thus, to properly determine the dose to be used in this study, uterus enlargement of rats was chosen as a marker of ZEA toxicity in a dose-range finding study (data not shown). Surprisingly, contrary to the anabolic effect of ZEA exposure reported [2,30], the first signs of toxicity observed in this dose-range experiment were weight loss and lower food intake in those animals exposed to the mycotoxin. Hence, 3.0 mg/kg of ZEA, the lowest dose that caused both an increase in the uterus’ relative weight and a decrease in food intake resulting in a lower weight gain, was selected for this immunotoxic study. 

In addition, it is well established that the immune system is highly susceptible to an inadequate supply of either macronutrients or selected micronutrients [31]. Therefore, once determined the dose and the inclusion of a pair-fed group (PF-group) to exclude a possible indirect effect of undernourishment, ovariectomized rats were subjected to the follow immune evaluation: heaematologic parameters, lymphoid organs study, and acquired and innate immune responses.

### 2.2. Food Consumption, Body Weight Gain and Haematologic Parameters

During the entire experimental period, no leftover food or gnawed feed were found into the cages of any of the ovariectomized rats. None of the rats showed signs of toxicity, such as soft feces, depression or other neurological manifestations, or death. However, the females from the ZEA group showed a significant reduction in their food consumption throughout the experimental period compared to rats from the control group (*p* < 0.01). Nevertheless, only the rats treated with the mycotoxin showed a significant decrease in body weight gain when compared to both groups of untreated rats. With respect to the haematologic analyses, no significant differences were observed among the groups for any of the parameters evaluated (Table 1). The histology of the non-lymphoid organs showed no significant morphological changes.

**Table 1:** Effects of zearalenone on total food consumption, body weight gain and haematologic parameters of ovariectomized rats.

Groups	Food consumption (g)	Body weight gain (g)	RBC (×10^6^/mm^3^)	WBC (×10^3^/mm^3^)	Ht (%)	Hg (g/dL)	MCH (pg)	MCV (mm^3^)
Control	448.2 ± 26.1	41.8 ± 13.1	7.2 ± 0.2	6.1 ± 0.5	40.8 ± 1.3	15.0 ± 0.3	20.9 ± 0.4	56.8 ± 0.5
3.0 mg/kg	390.3 ± 35.7 ***	22.7 ± 13.9 **^,†^	8.2 ±0.3	5.8 ± 0.6	45.5 ± 1.9	16.3 ± 0.6	19.9 ± 0.3	55.2 ± 0.5
Pair-fed ^a^	-	35.1 ± 7.8	7.8 ± 0.2	5.8 ± 0.4	44.0 ± 1.3	15.8 ± 0.4	20.3 ± 0.1	57.0 ± 0.4

Notes: RBC: red blood cells; WBC: white blood cells, Ht: haematocrit; Hg: haemoglobin; MCH: mean corpuscular haemoglobin; MCV: mean corpuscular volume; ** *p* < 0.01 and *** *p* < 0.001 *versus* the control group; **^†^** *p* < 0.05 *versus* the pair-fed group. The data are expressed as the means ± S.D; ^a^ Rats from the pair-fed group received an amount of diet equivalent to that consumed by rats from the group treated with 3.0 mg/kg of zearalenone.

### 2.3. Evaluation of Lymphoid Organs and Their Cells

**Figure 1:**
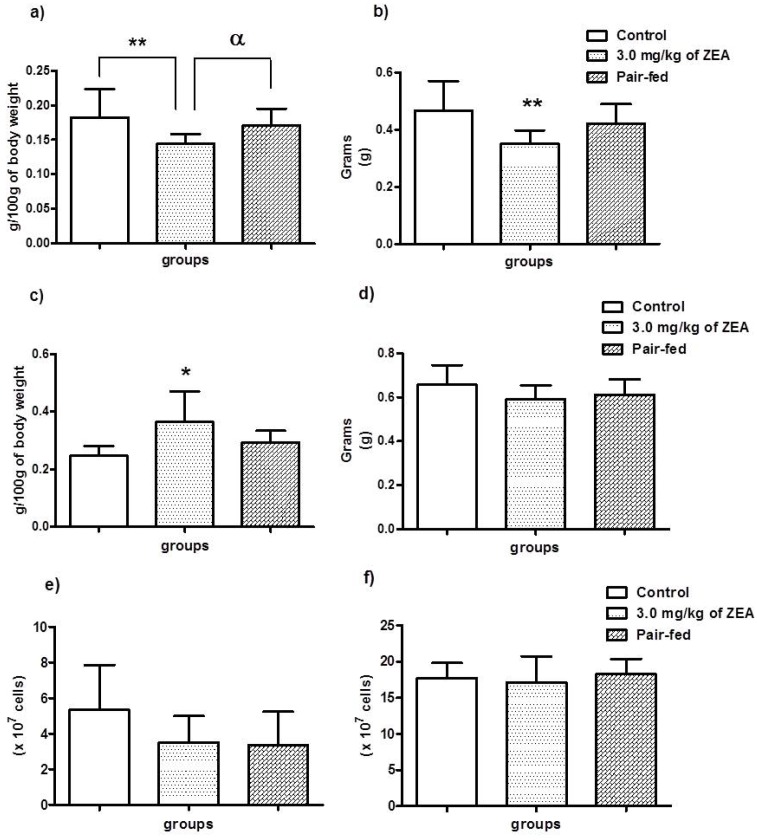
Thymus (**a**) and spleen (**c**) relative weight, thymus (**b**) and spleen (**d**) wet weight, splenocytes (**e**) and bone marrow (**f**) cellularity of female ovariectomized rats treated or not with 3.0 mg/kg of zearalenone for 28 days by gavage. The data are expressed as the means ± S.D. * *p* < 0.05; ** *p* < 0.01 *versus* the control group; ^α^ *p* < 0.05 *versus* the pair-fed group.

The thymus and spleen of each rat in all groups were weighed. The statistical analysis revealed that both organs were altered; however, while the relative weight (*p* < 0.01) and wet weight (*p* < 0.01) of the thymuses were reduced relative to both untreated groups, the relative weights of the spleens were increased when compared to the control group (*p* < 0.05), as shown in Figure 1a–c, respectively. However, statistical significance was lost when the wet spleen weights of the ZEA-treated group were compared to the control group (Figure 1d). Despite the alterations in the organ weights, no statistically significant differences were observed in the splenocyte numbers or in the bone marrow cellularity of the treated and untreated animals (Figure 1e and f, respectively). 

**Figure 2:**
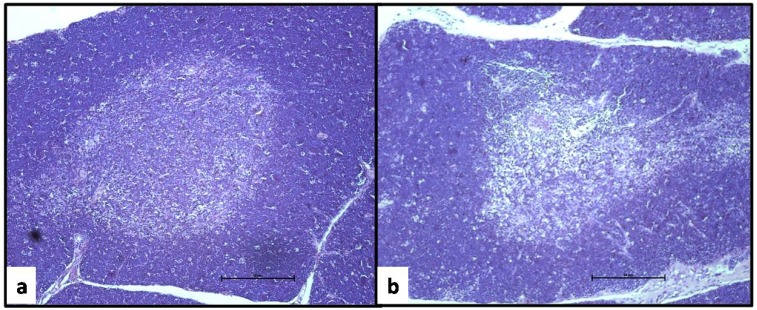
Thymus sections of an ovariectomized rat from the control group (**a**) and from a rat treated with 3.0 mg/kg of zearalenone by gavage for 28 days (**b**). Note the cellular depletion in both the cortex and medulla areas of the thymus of the rat treated with the mycotoxin (H.E., 4×).

Figure 2 shows the micrographs of the thymus sections of a rat from the control group (a) and the group treated with 3.0 mg/kg of ZEA (b) after 28 days. Although there were no differences in the cortex/medulla ratio (data not shown), the cellular depletion in both areas of the thymus, mainly in the medullar zone, should be noted. The thymus samples of the rats from PF group did not show these features.

Thymocyte phenotyping revealed that the percentages of mature T cells in the ovariectomized rats treated with ZEA were significantly different from the controls; however, while CD3^+^CD8^+^ cells (T cytotoxic cells) were increased (*p* < 0.05), CD3^+^CD4^+^ cells (T helper cells) were decreased (*p* < 0.05) in the thymuses of these rats compared to both groups of untreated animals. Conversely, when splenocyte phenotyping was performed, only IgM^+^CD45R^+^ cells (B cells) were significantly diminished (*p* < 0.01) in the rats treated with the mycotoxin relative to the animals from the untreated groups (Table 2). However, no differences were observed in the TCD4^+^/TCD8^+^ cells (data not shown).

**Table 2:** Effects of zearalenone on thymus and spleen lymphocyte phenotypes of ovariectomized rats.

Treatment (*n* = 10/group)	Thymus (% total lymphocytes)				Spleen (% total lymphocytes)		
	CD3^-^CD4^+^CD8^+^	CD3^+^CD4^+^CD8^+^	CD3^+^CD4^+^CD8^-^	CD3^+^CD4^-^CD8^+^	IgM^+^CD45R^+^	CD3^+^CD4^+^	CD3^+^CD8^+^
Control	87.9 ± 6.2	13.4 ± 3.0	67.0 ± 4.4	10.0 ± 2.0	29.5 ± 2.9	43.7 ± 3.1	43.9 ± 3.4
3.0 mg/kg	90.2 ± 1.6	17.7 ± 3.7	59.5 ± 4.3*	12.2 ± 1.2*	22.1 ± 3.4*	43.4 ± 7.6	44.3 ± 6.3
Pair-fed^a^	91.0 ± 1.4	18.1 ± 3.9	61.6 ± 5.5	11.7 ± 1.2	29.1 ± 3.2	41.6 ± 4.4	44.7 ± 3.8

Notes: ^a^ Rats from the pair-fed group received an amount of diet equivalent to that consumed by rats from the group treated with 3.0 mg/kg of zearalenone;* *p* < 0.05 *versus* control group. The data are expressed as the means ± S.D.

### 2.4. Acquire Immune Responses

**Figure 3:**
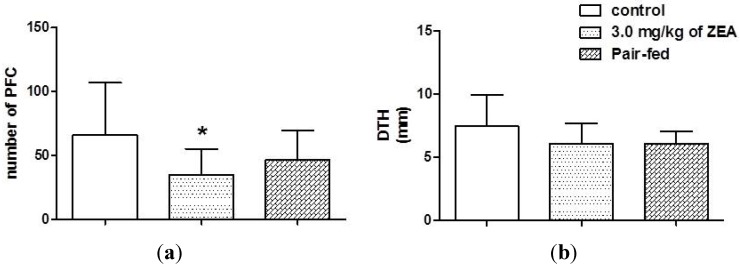
The effect of zearalenone exposure on plaque-forming cells (**a**), and delayed-type hypersensitive response (**b**) of rats treated or not with 3.0 mg/kg of zearalenone by gavage for 28 days. The data are expressed as the means ± S.D. * *p* < 0.05 *versus* the control group.

Figure 3a shows the plaque-forming cells (PFCs), a humoral assay that detected B cells producing immunoglobulin (IgM) antibody to sheep erythrocytes (SRBC), of rats treated for 28 days with ZEA by gavage and those of the controls. In spite of the reduction in SRBC-specific antibody production in both the ZEA and PF groups, only the animals in the ZEA group showed a statistically significant reduction in the PFC numbers compared to both untreated groups of female rats. On the other hand, no statistically significant differences were observed in the delayed-type hypersensitivity responses (DTH) among the three groups of rats (Figure 3b).

### 2.5. Innate Immunity: Macrophage Activity

**Figure 4:**
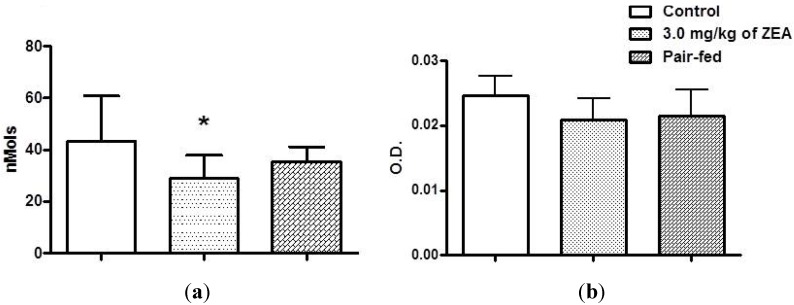
The effect of zearalenone exposure on spontaneous hydrogen peroxide release (**a**) and spontaneous nitric oxide production (**b**) by resident peritoneal macrophages of rats treated or not with 3.0 mg/kg of zearalenone by gavage for 28 days. The data are expressed as the means ± S.D.* *p* < 0.05 *versus* the control group.

Despite the fact that ZEA exposure did not affect the spreading and phagocytic properties of the peritoneal macrophages evaluated (data not shown), a reduction in hydrogen peroxide production by macrophages from both the ZEA and PF groups was observed. However, only the inflammatory cells from the rats treated with the mycotoxin showed a statistically significant reduction in this response (*p* < 0.05) relative to both untreated groups (Figure 4a). In contrast, the statistical analysis did not reveal any differences in nitric oxide (NO) production among the groups of rats (Figure 4b).

## 3. Discussion

We clearly show a significant impairment in the body weight gain in rats treated with 3.0 mg/kg of ZEA by gavage for 28 days: the weight gain values were approximately 50% lower than those of the control animals. On the other hand, despite the fact that the PF animals received the same amount of food as that consumed by rats from the ZEA group, the weight gain of these animals decreased by only 15% compared to the females of the control group.

Estrogens are known to have inhibitory effects on body weight gain in animal models [32,33]. This is consistent with the observations that ovariectomized rats (*i.e.*, in absence of circulating estrogen) are hyperphagic and gain more weight [34,35]. Thus, the pronounced decrease in the body weight of ZEA-treated rats observed in the present study may be a direct effect of the estrogenic property of the mycotoxin.

The hypothalamus, specifically the arcuate nucleus, is the area of the central nervous system (CNS) in which food intake is controlled. In addition, the ventromedial hypothalamus controls energy expenditure and body weight homeostasis [36]. It is well known that ERα is abundantly expressed in these areas of the CNS. Given that ZEA acts on both ERα and ERβ [37], the reduced food intake and body weight gain observed in the present study could be related to direct effects of the mycotoxin on the control of food intake and energy balance. However, reduced food intake alone is not sufficient for this effect, as demonstrated by the results obtained from the PF group. 

There is a paucity of studies regarding the haematotoxicity of ZEA in rodents. Maaroufi *et al*. [38] reported coagulation dysfunction and some changes in blood parameters (haematocrit, MCV, platelets, and WBC) in rats intraperitoneally treated once with 1.5, 3.0, and 5.0 mg/kg of ZEA. Similar blood parameter changes were reported in another study in which mice were treated orally with a single dose of 40 mg/kg or more [39]. However, we did not observe any haematoxic effects in the ZEA-treated rats. Thus, the previously reported alterations of the blood parameters in ZEA-exposed animals could be related to the route of exposure (parenteral × oral route) and/or the high doses of the mycotoxin.

The ability of estrogen to influence thymic atrophy has been recognized for many years. In particular, pregnancy is a period in which serum estrogen levels are extremely high, and the size of the thymus decreases dramatically during pregnancy [40,41]. However, as stated above, the immune system is highly susceptible to malnutrition, and the atrophic thymus works as a “barometer” of undernourishment [42,43]. The PF group was important for determining that the specific effects of ZEA exceeded this compound’s effects on food intake, as demonstrated by the fact that the thymus wet weight, as well as the thymus relative weight in the rats treated with the mycotoxin, were significantly reduced relative to both untreated groups. Interestingly, Kawashima and colleagues [44] showed that the stromal cells of the thymus have three-fold higher ER levels than the T-cell fraction. Thus, compounds that act on these receptors could impair the thymus; however, the mechanism by which thymic atrophy occurs is still not known. These results indicate that similar to other estrogens and some endocrine disruptors [20,45,46], ZEA also has immunotoxic effects on the thymus.

Unlike the thymus, which is gradually replaced by fat tissue in adulthood, the spleen is not affected by the animal nutritional status and/or body weight, as it does not contain adipocytes [47]. As no infections, extramedular myeloproliferative disorders or hepatic disease that could result in spleen enlargement were observed [48], we can conclude that the increasing spleen relative weight in this study was an artifact of the methodology. Consistent with this hypothesis, the data indicated that the spleen wet weights and the splenocyte counts were not different among the groups of rats. 

Despite the lack of differences among the splenocyte counts in the three groups, the ZEA treatment caused statistically significant differences in the subsets of lymphocyte in both lymphoid organs evaluated: the spleen and thymus. ERs are expressed in most immune cells, including B cells, T cells, and thymocytes [49,50]. In a study performed by Phiel *et al*. [51], CD4^+^ T cells were shown to express much higher levels of ERα than B cells, which in turn express more ERβ than CD4^+^ T cells. In contrast, CD8^+^ T cells express these two receptors at approximately equivalent levels. Thus, it is possible that ZEA binding to these ERs could promote changes in the maturation of thymocytes to mature T cells in the thymus, leading to the associated atrophy, and reduced size and cellularity observed in the thymus histopathology. Consistent with this concept, a study performed by Zoller and Kersh [52] showed that elevated estradiol levels inhibit thymocytes at multiple stages of development; therefore, one hypothesis is that this estrogenic mycotoxin acts similarly to other estrogenic compounds in the thymus. 

In addition, estrogens may also affect the B cell development pathway in the bone marrow through a variety of mechanisms, including inhibition and induction of apoptosis [53]. However, no differences on bone marrow cellularity were observed in the present study. Thus, the reduction in the mature B cell population of the spleens could be a direct effect of ZEA on these cells after leaving the bone marrow. In support of this mechanism, a study performed by Salah-Abbès *et al*. [24] showed that ZEA reduced the number of circulating B lymphocytes in the blood of mice. 

The effect of estrogen on the immune system had received much attention due to its immunomodulatory activity on cell-mediated responses and antibody production. As we mentioned previously, pregnancy, a period of high estrogen levels, affects the maternal immune system to permit fetal development, negatively modulating cell-mediated immunity and augmenting immunoglobulin secretion [20,54]. Thus, estrogen enhances the humoral immune responses. However, in this study, ZEA reduced the splenic plaque-forming cell response. Recently, a study performed with rats treated with 5.0 mg/kg of ZEA for 36 days revealed that ZEA alone (without immune challenge) can decrease the production of immunoglobulins [25]. In addition, an *in vitro* study with peripheral blood mononuclear cells (PBMC) of piglets also showed a decrease in immunoglobulin levels [26]. Thus, the inconsistent effects of estrogen and ZEA on humoral immune response could be related to receptor-specific effects. Specifically, ZEA is an agonist toward ERα and a mixed agonist-antagonist of ERβ [37], with possible full antagonism of the ERβ expressed by B cells. However, more studies should be conducted to confirm this hypothesis. 

Studies in which ovariectomized mice were treated with type II collagen to induce rheumatoid arthritis (RA) have provided a useful tool to better understand the impact of estrogen on this autoimmune disease. The condition is characterized by a systemic inflammatory disorder that reaches a peak of incidence in women during menopause, a physiological state during which the estrogen levels decrease [55]. In a study performed by Engdahl *et al*. [56], it was observed that ERα, but not ERβ, signalling promoted a significant improvement collagen-induced RA effects in the mice. Knowing that ZEA is a full agonist on ERα and this receptor is expressed in macrophages more than in monocytes [57], it could be hypothesized that ZEA, similar to estrogen, can suppress inflammatory response mediated by the release of hydrogen peroxide by peritoneal macrophages. Consistent with this concept, in an *in vitro* study in which piglet PBMCs were exposed to ZEA, the mycotoxin impaired the inflammatory response of these cells, revealing its suppressive effect [26]. However, in the present study, it is not clear why other phagocyte activities (phagocytosis and NO production) were not suppressed.

## 4. Experimental Section

### 4.1. Animals and Treatment Regimen

Ovariectomized female adult Wistar rats (10 wk of age) were obtained from our colony in the Department of Pathology, School of Veterinary Medicine and Animal Sciences, University of São Paulo. All rats were housed separately, received food and water *ad libitum* and were maintained under controlled conditions regarding temperature (22–25 °C), relative humidity (50%–65%) and lighting (12 h/12 h, light/dark cycle). Food consumption and body weight gain were measured every other day. The experiments were carried out in accord with the ethical principles for animal research adopted by the Bioethics Committee of the School of Veterinary Medicine and Animal Sciences. 

Three experimental groups were simultaneously used for all assays performed, and each group consisted of 10 animals. One group was treated with 3.0 mg/kg of body weight of ZEA dissolved in corn oil (ZEA group), one was treated with corn oil (control group), and the third group was an untreated PF group. The rats from the control and ZEA groups were treated orally by gavage daily for 28 days. As ZEA caused a reduction in food consumption, a pair-fed group (PF group) was included in this study to assess whether any observed immunotoxic effects were the result of a direct effect of the mycotoxin or decreased nutrient support. The PF group received an amount of food equivalent to that consumed daily by rats from ZEA group. Thus, food intake was identical in the PF and ZEA groups. Clinical examination of the animals’ condition was performed every other day; in addition, food consumption and body weight gain were evaluated.

### 4.2. Reagents

Lipopolysaccharide (LPS 0127:B8), peroxidase, phorbol-myristate acetate (PMA), and propidium iodide (PI) were purchased from Sigma Chemical Co. Foetal bovine serum (FBS), HEPES, RPMI culture medium-1640, and trypan blue were obtained from Gibco. Keyhole limpet haemocyanin (KLH) and ethylenediaminetetraacetic acid (EDTA) were obtained from Merck. Xylazine and ketamine were purchased from Virbac. FITC-labeled anti-CD3 (clone G4.18), PE-labeled anti-CD4 (clone OX-35), PerCP-labeled anti-CD8a (clone OX-8), PE-labeled anti-IgM (clone G53-238), FITC-labeled anti-CD45R (clone HIS24), and purified anti-CD32 (clone D34-485) Rat BD Fc Block™ antibodies and rat cytokine immunoassay kits were purchased from BD Pharmingen. ZEA (Lot#Z007, with purity greater than 99%) was purchased from Fermentek.

### 4.3. Haematology, Immune Organ and Lymphoid Cell Analysis

On day 29, all rats from the three experimental groups were intraperitoneally anaesthetized with xylazine and ketamine (5 and 50 mg/kg, respectively) to collect blood from the caudal vena cava. After euthanasia by cervical dislocation, the lymphoid organs (thymus and spleen) and bone marrow from the left femur were harvested to evaluate the cell numbers and the immunopathological effects of ZEA. Blood collection was performed with EDTA to permit evaluation of the haematological parameters using automated Horiba^®^ ABX equipment. 

For the lymphoid organ analyses, the thymus and spleen were removed from the euthanized rats and weighed. Half of each spleen and thymus was disrupted using two pieces of ground glass, and the red blood cells were removed by lysing with a sterile solution of 0.4% ammonium chloride, resulting in suspensions of single splenocytes and thymocytes in cold RPMI-1640 culture medium. The bone marrow cell suspensions were achieved by flushing the marrow cavity of the left femur of each rat with ice-cold RPMI-1640 medium using a sterile syringe with a 26-gauge needle. Viability was assessed using a trypan blue dye exclusion test, and cell numbers were determined using a haemocytometer. In addition, fragments of liver, spleen, thymus, lungs, and kidney were collected and fixed in 10% formalin, routinely embedded in paraffin, cut into 5-μm thick sections, and stained with haematoxylin and eosin (HE) for histopathology. The histological sections of the rat thymus were submitted to morphometric analysis in an Image-Pro Plus system, Version 4.5 (Media Cybernetics Inc., Bethesda, MD, USA), and the ratio of cortical to medullar areas (C/M) was determined and calculated using the formula:

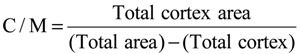
(1)


The spleen and thymus cell suspensions from each rat were resuspended in RPMI medium supplemented with 10% heat-inactivate fetal bovine serum, 100 U/mL penicillin, and 100 μg/mL streptomycin and incubated in 6-well plates for 2 h at 37 °C in a humidified 5% CO**_2_** incubator. After this period, the non-adherent cells were harvested, washed with RPMI, adjusted to 1 × 10^6^ cells and incubated with 0.5 μL BD rat Fc Block for 5 min to block the Fc-mediated adherence of antibodies prior to staining with the following specific antibodies: anti-CD3, anti-CD4 and anti-CD8 antibodies to detect the thymocytes and T lymphocytes and anti-IgM and anti-CD45R to detect the B lymphocytes. Then, the cells were incubated for 30 min at 4 °C in the dark. After washing to remove unbound antibody, the cells were resuspended in PBS for flow cytometric assessment using a FACSCaliburTM flow cytometer equipped with Cell Quest Pro^®^ software (Becton Dickinson (BD) Immunocytometry System). Data from a total of 10,000 target cells were collected by flow cytometry, and the results are expressed as percentages. The data were analyzed using FlowJo 7.2.2^®^ software (Tree Star Inc., Ashland, KY, USA).

### 4.4. Humoral Immune Response

On Day 21 of the regimen, the rats (*n* = 10/treatment group) were immunized by intraperitoneal (IP) injection of 2.0 × 10^9^ sheep red blood cells (SRBC) in 0.9% saline (normal saline). The Plaque-Forming Cell assay (PFC) was used to assess the status of the humoral immune response of each host. Briefly, on day 29, single-cell suspensions of the rat spleens (10^7^ cells/mL) were prepared in RPMI-1640 medium at 4 °C. The splenocyte suspensions, SRBC, and guinea pig complement were added to a 1.0 cm^2^ PFC well to obtain a final concentrations of 2.0 × 10^6^ splenocytes/mL, 7% SRBC, and 10% complement in a final volume of 50 μL; duplicate wells were prepared for each rat sample. The wells were then covered with glass slides (22 mm × 22 mm) and sealed with varnish. Each PFC well was incubated at 37 °C for 3 h before the number of lysate plaques produced by the 10^5^ splenocytes in the well was counted using a light microscope (40× magnification). The data are normalized and expressed as the total PFC by 10^6^ splenocytes.

### 4.5. Delayed Type Hypersensitivity (DTH)

The rats in the three experimental groups were sensitized with KLH as described by Exon *et al*. [58]. Briefly, KLH (5 mg/mL) was injected into the caudal tail fold in a 200 μL volume of sterile water on days 14 and 21. At the end of the experimental period (Day 28), all of the rats were challenged with heat-aggregated KLH (80 °C for 1 h) in 0.1 mL of saline (20 mg/mL). The challenge antigen was injected into one footpad, while the other footpad received sterile saline. Swelling was measured with a micrometer and calculated by subtracting the thickness of the saline-injected left footpad from that of the KLH-injected right footpad. Swelling was then plotted as the difference between the measurements.

### 4.6. Innate Immunity: Macrophage Activity

At the end of the experimental period, the activity of the peritoneal macrophages from the rats of the three experimental groups was evaluated using the protocols previously described by Rabinovitch and Destefano [59,60]. 

Briefly, the macrophages were obtained by lavage of the peritoneal cavity with 10 mL of cold PBS. The cells were centrifuged and resuspended in RPMI and adjusted to 2 × 10^6^ cells/mL. Duplicate aliquots of 200 μL of the cell suspension from each animal were then placed in multi-well (6 wells) tissue culture plates. To assess phagocytic activity, 1 mg of opsonized zymosan A solution (5.0 mg/mL) was added to each well, and the cells were then incubated for 60 min at 37 °C. After this period, the wells were rinsed with PBS to remove both un-ingested zymosan and any non-adherent cells. Thereafter, a total of 200 cells by well were analyzed and the macrophage spreading index (SI%) and phagocytic index (PI%) was calculated as follows: SI% or PI% = number of spreading macrophages or phagocytic cells (SI or PI) × 100 ÷ 200 adherent cells counted, where SI and PI are the number of spread cells or the number of cells that ingested ≥1 particle, respectively. Each well was examined twice; the mean of the four values obtained from the duplicate counts from two wells/rat was then used for the statistical analyses of SI and PI.

The spontaneous and phorbol myristate-acetate solution (PMA)-induced H2O2 release by macrophages was measured according to the method of Russo and collaborates [61]. The H_2_O_2_ concentration was calculated from the absorbance measurements as described by Pick and Mizel [62]. In addition, the concentrations of NO in supernatants of macrophages seeded in the presence or absence of LPS (100 ng/mL) for 24 h were measured using the Griess reagent [63]. Briefly, the Griess reagent was prepared by mixing an equal volume of 1% sulfanilamide in 5% phosphoric acid and 0.1% naphthylethylenediamide just prior to use. The Griess reagent (100 μL) was mixed with an equal volume of the cell supernatants. After incubation at room temperature for 10 min, the OD value was measured using a microplate reader (PowerWave HT, BioTek^®^) at 540 nm. All experiments for the NO measurements were performed in triplicate.

### 4.7. Statistical Analysis

Student’s t-test was used to compare two groups, and a one-way analysis of variance (ANOVA) followed by Tukey’s *post hoc* test was used for more than two groups, with the level of significance set at *p* < 0.05. However, if Bartlett’s test suggested that the differences among the S.D. values were significant, the Kruskal-Wallis test was employed, followed by Dunn’s Multiple Comparisons Test. The data are expressed as the means ± S.D.

## 5. Conclusions

Our results show that ZEA causes a decrease in body weight that is not solely related to the reduction of food consumption. Moreover, we verified that similar to estrogen, ZEA can modulate most aspects of immune responses and impair lymphoid organs, resulting in thymus atrophy; the mycotoxin can alter thymus and spleen lymphocyte phenotypes and decrease peroxide production by peritoneal macrophages; and unlike estrogen, ZEA can impair the T cell-dependent humoral immune response.
